# The *Schistosoma mansoni* phylome: using evolutionary genomics to gain insight into a parasite’s biology

**DOI:** 10.1186/1471-2164-13-617

**Published:** 2012-11-13

**Authors:** Larissa Lopes Silva, Marina Marcet-Houben, Laila Alves Nahum, Adhemar Zerlotini, Toni Gabaldón, Guilherme Oliveira

**Affiliations:** 1 Grupo de Genômica e Biologia Computacional, Centro de Pesquisas René Rachou. Instituto Nacional de Ciência e Tecnologia em Doenças Tropicais. Fundação Oswaldo Cruz - FIOCRUZ, Belo Horizonte, MG, 30190-002, Brazil; 2 Centro de Excelência em Bioinformática, Fundação Oswaldo Cruz – FIOCRUZ, Belo Horizonte, MG, Brazil; 3 Instituto de Ciências Biológicas, Universidade Federal de Minas Gerais – UFMG, Belo Horizonte, MG, Brazil; 4 Bioinformatics and Genomics Programme, Centre for Genomic Regulation (CRG), Dr. Aiguader, 88, 08003, Barcelona, Spain; 5 Universitat Pompeu Fabra (UPF), 08003, Barcelona, Spain; 6 Faculdade Infórium de Tecnologia, Belo Horizonte, MG, 30130-180, Brazil; 7 Laboratório Multiusuário de Bioinformática, Embrapa Informática Agropecuária, Campinas, São Paulo, Brazil

**Keywords:** Phylogenomics, Maximum likelihood analysis, Homology prediction, Functional annotation, Paralogous families, Parasite genomics, Schistosomiasis

## Abstract

**Background:**

*Schistosoma mansoni* is one of the causative agents of schistosomiasis, a neglected tropical disease that affects about 237 million people worldwide. Despite recent efforts, we still lack a general understanding of the relevant host-parasite interactions, and the possible treatments are limited by the emergence of resistant strains and the absence of a vaccine. The *S. mansoni* genome was completely sequenced and still under continuous annotation. Nevertheless, more than 45% of the encoded proteins remain without experimental characterization or even functional prediction. To improve our knowledge regarding the biology of this parasite, we conducted a proteome-wide evolutionary analysis to provide a broad view of the *S. mansoni*’s proteome evolution and to improve its functional annotation.

**Results:**

Using a phylogenomic approach, we reconstructed the *S. mansoni* phylome, which comprises the evolutionary histories of all parasite proteins and their homologs across 12 other organisms. The analysis of a total of 7,964 phylogenies allowed a deeper understanding of genomic complexity and evolutionary adaptations to a parasitic lifestyle. In particular, the identification of lineage-specific gene duplications pointed to the diversification of several protein families that are relevant for host-parasite interaction, including proteases, tetraspanins, fucosyltransferases, venom allergen-like proteins, and tegumental-allergen-like proteins. In addition to the evolutionary knowledge, the phylome data enabled us to automatically re-annotate 3,451 proteins through a phylogenetic-based approach rather than solely sequence similarity searches. To allow further exploitation of this valuable data, all information has been made available at PhylomeDB (http://www.phylomedb.org).

**Conclusions:**

In this study, we used an evolutionary approach to assess *S. mansoni* parasite biology, improve genome/proteome functional annotation, and provide insights into host-parasite interactions. Taking advantage of a proteome-wide perspective rather than focusing on individual proteins, we identified that this parasite has experienced specific gene duplication events, particularly affecting genes that are potentially related to the parasitic lifestyle. These innovations may be related to the mechanisms that protect *S. mansoni* against host immune responses being important adaptations for the parasite survival in a potentially hostile environment. Continuing this work, a comparative analysis involving genomic, transcriptomic, and proteomic data from other helminth parasites, other parasites, and vectors will supply more information regarding parasite’s biology as well as host-parasite interactions.

## Background

*Schistosoma mansoni, S. haematobium, and S. japonicum* (Platyhelminthes: Trematoda) are the main causative agents of human schistosomiasis, a neglected tropical disease that is endemic in 77 countries where more than 237 million people require preventive chemotherapy and other 779 million live in areas of risk of infection [1-4]. The genomes of these parasites have been recently published providing insights into parasite’s development, infection, and host-parasite interactions [5-7]. However, even with the progress made over the last years, schistosomiasis control depends primarily on the treatment of infected patients with Praziquantel^®^, the only drug available for mass treatment (e.g. [5,8,9]). Drawbacks of this drug are that it does not prevent against reinfection and its effectiveness varies depending on several factors such as the parasite’s gender, developmental stage, and the time of infection. Furthermore, Praziquantel^®^-resistant parasites have been found both in the laboratory and in the field, thus increasing the urgent need for new effective drugs and vaccines [10-13].

*Schistosoma mansoni* infects 7.1 million people in America, 95% of which in Brazil, and 54 million people in Sub-Saharan Africa causing intestinal and hepatosplenic schistosomiasis [14,15]. The *S. mansoni* genome sequencing data was published in 2009 and a new version was recently released [5,16]. The improved genome has 364.5 megabases (Mb) assembled in 885 scaffolds, half of which are represented in scaffolds greater than 2 kilobases [16]. A total of 10,852 genes were identified, encoding over 11,000 proteins, 45% of which remain without known or predicted function [5,16,17]. 81% of the genome was assembled onto the parasite’s chromosomes, providing a partial genetic map [16,18]. The availability of genomic data offers new opportunities for innovation in the control of schistosomiasis, by providing information that allows for the identification of novel drug targets and vaccine candidates through a system-wide perspective [5,19,20].

Making accurate functional predictions for genes or proteins is a key step in every genome sequencing project. However, on average, 30 to 50% of the predicted proteome remains uncharacterized while for the remaining set only general predictions are made. To deal with the gap between the rapid progress in genome sequencing and experimental characterization of genes and gene products, computational methods have been developed [21-23]. Two main approaches are generally used for functional prediction of genes and their products: one based on sequence similarity searches and another on phylogenetic analysis.

Owing to the computational cost and complexity of large scale phylogenetic analysis, the accurate identification of orthology relationships remains a challenge in comparative genomics and most of the orthology prediction methods rely on similarity-based search (e.g. BLAST [24], OrthoMCL [25], InParanoid [26]). In these cases, functional prediction is obtained based on the transfer of information from the most similar sequences in the database to the gene or protein of interest (e.g. [24]). However, several limitations are associated with this method, mainly the lack of a straightforward relationship between sequence similarity and protein function [21,27-29]. Since this approach is fast, simple, and can be automated to analyze thousands of genes, it has been used frequently to predict functional products encoded by newly sequenced genomes. Over the last years this practice has generated systematic errors, the extent of which is not completely known [22,27-32].

In an attempt to improve the accuracy of functional prediction at a large scale, phylogenetic methods may be applied [33,34]. The advantage of such methods is that they focus on the evolutionary history of genes rather than merely on their sequence similarity [30,35,36]. Ideally, functional transfer in the genomic context or for specific genes/proteins should be performed only when there is any experimental evidence for those used as source of information. However, in databases as UniProt, only 3% of proteins have experimental support for their annotations [28]. To deal with the absence of experimental support for most part of the available proteomes, transfer of functional annotation aiming to provide hints regarding the gene/protein function needs to follow strict requirements to avoid, as much as possible, misclassifications. In the last decade, the publication of a large number of genomic and proteomic data and the development of faster and powerful computers, new software, and automated pipelines have allowed for the reconstruction of phylogenetic trees of the complete set of proteins encoded in a genome – the so called phylome [37].

The phylome data may give a broad view of the evolution of an organism, since it comprises the phylogenies of all proteins encoded in its genome [37]. Most notably, a phylome can be used to detect specific evolutionary scenarios, to quantify the fraction of individual phylogenies whose topologies are consistent with a given hypothesis, and to improve functional annotation of proteins and biological systems [38,39]. Furthermore, comparing genomes or proteomes through an evolutionary perspective may provide insights to the understanding of the metabolism, physiology, pathogenicity, and the adaptation to a particular life style of organisms. In this context, the availability of *S. mansoni* genomic data provides the opportunity to study this parasite from a genome-wide perspective rather than from individual gene or protein analyses.

Taking advantage of the benefits provided by a genome-wide approach combined with an evolutionary perspective, we reconstructed the *S. mansoni* phylome with the goals of i) gaining insight into lineage-specific evolutionary events potentially related to the parasitic lifestyle, and ii) improving the functional annotation of the genome/proteome.

Phylogenetic techniques used in the present work included multiple sequence alignment [40-43] alignment trimming [44], neighbor-joining tree building [45], evolutionary model testing, and maximum likelihood analysis [46]. The resulting phylome data contains 7,964 protein phylogenetic trees, covering the analysis of 11,763 *S. mansoni* proteins and their homologs in 12 other organisms, out of which we identified evolutionary events and homology relationships. The results provided useful information about the parasite’s genome evolution such as the identification of gene duplication events and expanded protein families such as proteases, tetraspanins, fucosyltransferases, venom allergen-like proteins (also called as SmVAL or SCP-like), tegumental-allergen-like proteins (SmTAL), among others. Altogether, the results obtained are likely to pave the way for a better understanding of the parasite’s biology including host-parasite interactions. This, in turn will accelerate the search for new drugs and vaccine directed toward the control and eradication of schistosomiasis.

## Results and discussion

### Reconstruction of the *S. mansoni* phylome

The *S. mansoni* phylome reconstructed in this work was derived from the comparative analysis of all proteins encoded in the parasite genome (predicted proteome) and their homologs in 12 other eukaryotic proteomes whose genomes were completely sequenced (Table 1). The set of selected species is particularly rich in metazoans (11 species), including ten invertebrates, one tunicate, and one vertebrate. One choanoflagellate*, Monosiga brevicollis*, was included as outgroup of the phylogenetic reconstruction. The metazoan species selected represent important evolutionary innovations, e.g. the origin of the third germ layer, the development of organs, systems, complex patterns of communication, and the emergence of the adaptive immune system, making this dataset set especially suitable for addressing the evolutionary innovations in *S. mansoni* in the context of metazoan evolution.

**Table 1:** **Proteomes selected for the *S. mansoni *phylome reconstruction**

**Scientific Name**	**UniProt Species Code**^**1**^	**TaxID**^**2**^	**Proteins**^**3**^	**Source**^**4**^	**Download**
*Monosiga brevicollis*	MONBE	81824	9,170	JGI	2011-06-01
*Ciona Intestinalis*	CIOIN	7719	14,048	UniProt Reference Proteomes	2011-07-09
*Nematostella vectensis*	NEMVE	45351	24,424	UniProt Reference Proteomes	2011-07-09
*Schistosoma haematobium*	SCHHA	6185	12,767	SchistoDB	2012-03-09
*Schistosoma mansoni*	SCHMA	6183	11,103	SchistoDB	2012-03-09
*Schistosoma japonicum*	SCHJA	6182	12,636	SchistoDB	2012-03-09
*Caenorhabditis elegans*	CAEEL	6239	19,758	UniProt Reference Proteomes	2011-07-09
*Ascaris suum*	ASCSU	6253	18,430	WormBase	2012-03-09
*Brugia malayi*	BRUMA	6279	19,916	WormBase	2012-03-09
*Trichinella spiralis*	TRISP	6334	15,878	WormBase	2012-03-09
*Drosphila melanogaster*	DROME	7227	11,794	FlyBase	2011-09-13
*Tribolium castaneum*	TRICA	7070	16,533	BeetleBASE - HGSC	2011-12-16
*Homo sapiens*	HUMAN	9606	20,965	UniProt Reference Proteomes	2011-07-09

1 - Code assigned to each species in the *S. mansoni* phylome. 2 - Taxonomic identifier at NCBI (TaxID). 3 - Number of proteins analyzed per species. 4 - Database from which the protein data were retrieved.

**Figure 1:**
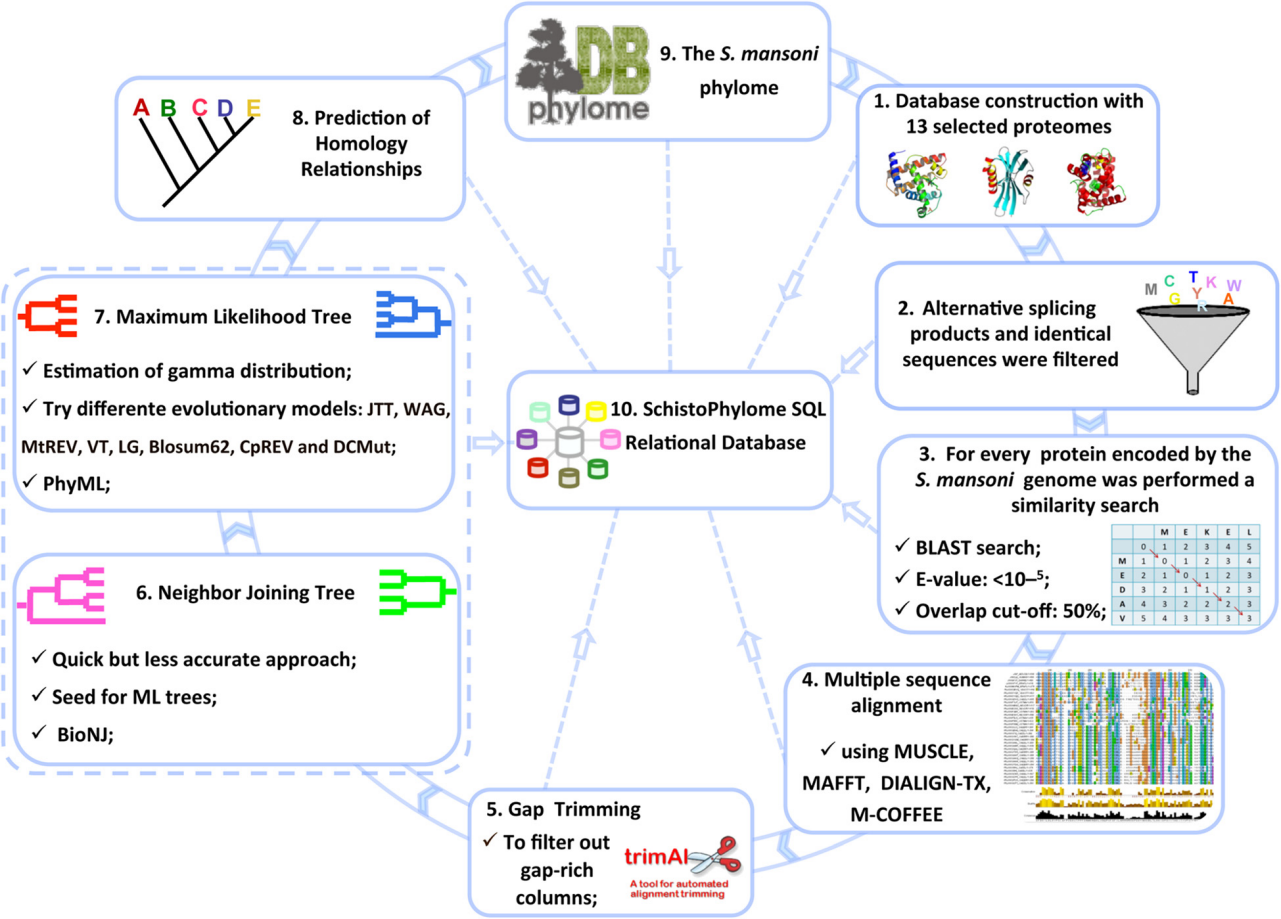
**Pipeline used to reconstruct and analyze the *S. mansoni *phylome.** Each protein sequence encoded in the parasite genome was compared against a database of proteins from other 12 fully sequenced eukaryotic proteomes (Table 1) to select putative homologous proteins. Groups of potential homologs were aligned and subsequently trimmed to remove gap-rich regions. The refined alignment was used to build a NJ tree, which was then used as a “seed” tree to perform a ML likelihood analysis as implemented in PhyML. In the ML analysis, up to five different evolutionary models were tested and the model best fitting to the data was determined by the Akaike Information Criterion (AIC). Different algorithms were used to identify homology relationships and lineage-specific duplications. To extract and interpret the large data set obtained a Structured Query Language (SQL) relational database was built. This database was the main resource for data mining in this work. Adapted from [39].

To perform the phylogenetic analyses, we applied an automated pipeline similar to the one used for the human phylome project [39]. This pipeline is illustrated here (Figure 1). The resulting alignments, phylogenies, and orthology predictions can be accessed at PhylomeDB [47] (http://phylomedb.org).

Using this phylogenomic approach, we analyzed 11,763 *S. mansoni* proteins and obtained 7,964 phylogenetic trees covering 70% of the parasite’s proteome. This coverage is remarkably similar to that of other phylome data of newly sequenced genomes such as that of the pea aphid *Acyrthosiphon pisum* (67%) [38].

The absence of trees for the remaining 3,490 proteins is either due to a possible high degree of divergence between the *S. mansoni* proteins and their homologs in the other selected species, an indication of the uniqueness of the parasite’s proteome, or it reflects the presence of errors in gene models. Out of the 7,964 phylogenetic trees, 3.038 (38%) correspond to trees with “seed” proteins with a completely unknown function and without any GO [48] assignment in SchistoDB [17].

### Phylogeny-based orthology prediction

In order to create a complete list of orthology and paralogy relationships among *S. mansoni* proteins and those encoded in the other eukaryotic proteomes included in this work, we analyzed the parasite’s phylome using a *species-overlap* algorithm as previously described [39]. The comprehensive catalogue of phylogeny-based orthology and paralogy relationships among *S. mansoni* and other species was made publicly available at PhylomeDB [47].

**Figure 2:**
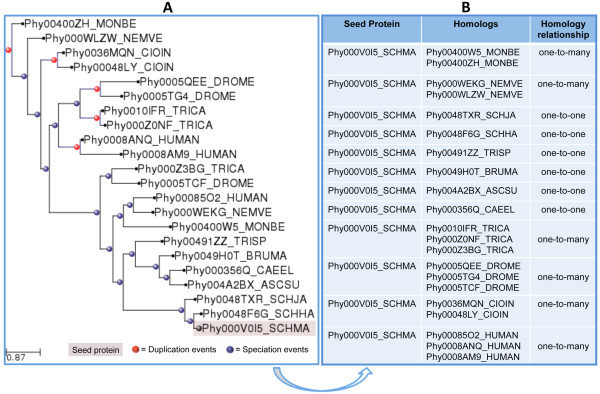
**Homology relationships and evolutionary events inferred from the analysis of a *S. mansoni *protein.A**) Phylogenetic tree reconstructed for the parasite “seed” protein Phy000V0I5_SCHMA (Smp_175750). **B**) Homology relationships identified between the “seed” protein and its homologs in the other species.

Owing to the increasing rate at which new fully sequenced genomes are released, the accumulation of genomic and proteomic data has been much higher than the rates at which genes or proteins are experimentally characterized. Aiming at producing a high confidence set of functional predictions for *S. mansoni* proteins, we used the evolutionary relationships as inferred from phylogenetic trees to obtain subsets of one-to-one (single homolog in *S. mansoni* and in other species) homology relationships among *S. mansoni* proteins and the homologs from other species included in the present study (Figure 2).

By using such phylogeny-based approach, we transferred 10,175 functional annotations (GO terms [48]) to 3,451 *S. mansoni* proteins, from which 790 (7% of the parasite’s proteome) were previously annotated as “hypothetical protein”, corresponding to proteins whose function had not been predicted or experimentally tested before (Additional file 1 Table S1). The transfer was performed from each ortholog with known function in the selected taxa to the *S. mansoni* “seed” protein. For the other proteins that already had any functional prediction, the annotation was confirmed or improved. Consequently, a “seed” protein could receive more than one functional description. In these cases, all functional annotations were maintained allowing the user to choose the closest related transferred functional annotation, those that came from model organisms, or even to create a consensus based on all of them.

To validate the applied methodology, we retrieved reviewed *S. mansoni* proteins from UniProt [49], including experimentally confirmed ones, to evaluate the annotation transferred by the phylogenomic approach. The functional annotations performed by PhylomeDB correspond to known functions in the aforementioned database (Additional file 1 Table S2). Even though the BLAST search may detect distant homologs with additional domains, our subsequent phylogenetic reconstruction and our selection of orthologs will select those orthologs that are likely to have similar domain architecture. This is an additional reason why an orthology-based annotation is preferred over sequence similarity searches, since orthologs as compared to paralogs have a higher tendency to share a similar domain architecture [50].

**Figure 3:**
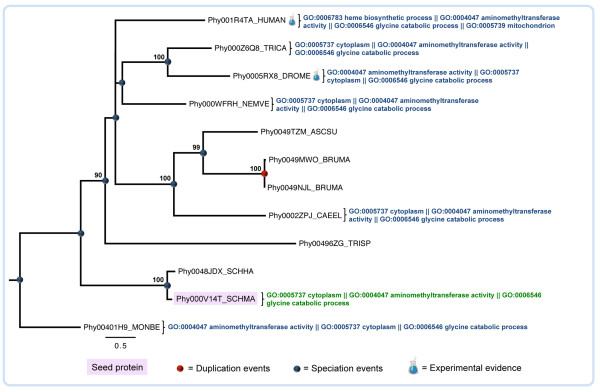
**Example of functional prediction based on phylogenetic analysis.** The protein sequences are represented by the internal identifier in PhylomeDB. Relationships among the parasite Phy000V14T_SCHMA “seed” protein (Smp_170950) and its homologs in other species (Table 1) as inferred by maximum likelihood method implemented in PhyML. Support values were computed by approximate likelihood ratio test (aLTR). Curly brackets hold Gene Ontology (GO) terms for proteins in this dataset.

Although less reliable than those based on one-to-one orthology relationships, annotation transfer based on more complex subsets (one-to-many, many-to-one, or many-to-many) may provide important hints to predict the biological function of *S. mansoni* proteins. However, in these cases, one or more genes are co-orthologous to a set of genes in another genome due to lineage-specific duplication(s) that can be associated with functional shifts, affecting the reliability of the functional transfer [38,51]. An example of a one-to-one transfer from a *Drosophila melanogaster* protein to a *S. mansoni* protein comes from the phylogenetic reconstruction of the Phy000V14T_SCHMA (Smp_170950) protein, potentially related to the glycine cleavage system, and its homologs in the selected species (Figure 3). The analysis of this tree resulted in six transfers of functional annotation from homologous proteins to the *S. mansoni* “seed” protein. The GO terms in all six functional annotations are related to aminomethyltransferase activity and glycine catabolic process providing further support for the annotation transfer. In this example, to illustrate a case of a one-to-one transfer, we chose the functional annotation transferred from *Drosophila melanogaster* once, according to the information available in UniProt [49], it is one of the orthologs with known function and experimental validation. Tags for homologous sequences with experimental validation are not available in PhylomeDB [47]. However, links to UniProt [49] and other databases are provided.

To explore the benefits offered by comparative genomics in order to improve functional annotation of genes and gene products, it is also necessary to consider the limitations involved in this approach. Although it is generally accepted that functional annotation through orthology, rather than just homology relationship, constitutes one of the most promising annotation approaches, these surveys are designed to provide predictions regarding the likely protein function, but it does not substitute experimental confirmation [36,52]. Functional diversity is often associated with significant divergence at the sequence level, but high levels of identity do not ensure that two or more proteins perform the same function, since subtle changes in active sites are able to completely change the protein function [53].

As we previously mentioned, evolutionary analysis involving fully sequenced genomes/proteomes remains a challenge. Although the tools here applied were not originally designed for large scale phylogenetic analysis, we adapted them to work on a large scale, since we strongly believe that a system-wide perspective on evolutionary processes can greatly improve the understanding on how genomes came to be and what evolutionary process took them there. Functional prediction as described in the present work could be used as a starting point for future projects, prioritizing the selection of certain genes or proteins for new experimental studies.

### Detection of gene duplications in *S. mansoni*

An additional advantage of the phylogeny-based approach is that it readily provides a collection of gene evolutionary histories that can be mined for particular events. Since gene duplication is considered one of the main mechanisms for functional innovation and diversification [54], we explored the *S. mansoni* phylome to identify protein families that have been specifically expanded in this lineage, since its diversification from the other sequenced metazoans. We used the above-mentioned *species-overlap* algorithm that identifies duplication nodes and also provides clues of the relative dating of the duplication event [39,55].

Such analysis revealed that in 3,051 reconstructed phylogenetic trees there is at least one paralog connected to the “seed” protein through a duplication node (Additional file 1 Table S3). Among these, 211 phylogenies show lineage-specific duplications in the three *Schistosoma* species in comparison with the other taxa. These expansions are small-to-moderate in size, resulting in a total of two to ten paralogs, and include some of the most significant expansions as discussed below.

The inclusion of *S. haematobium* and *S. japonicum* proteomes gave us a high resolution within *Schistosoma* genus and allowed us to make comparisons across this taxon. In general, the expansions observed in *S. mansoni* can also be observed in the other two *Schistosoma* species, although with variable number of paralogs in each species. As previously observed by evolutionary relationships, cytogenetic data, and syntenic analyses, the present study shows that *S. mansoni* is more closely related to *S. haematobium* than to the *S. japonicum*[56-59]. Moreover, 170 evolutionary trees have only *S. mansoni* and *S. haematobium* proteins, while only six phylogenies have solely *S. mansoni* and *S. japonicum* proteins. Meanwhile, most of the homologous proteins shared by *S. mansoni* and *S. haematobium* are annotated as “hypothetical protein” and do not have any predicted function or significant hits with known proteins in public databases as UniProt [49], Pfam [60], or non-redundant (nr) NCBI database (ftp://ftp.ncbi.nih.gov/blast/db).

A small number of phylogenetic trees (1,45%) had only sequences of *S. mansoni*. These could be the result of very recent duplication events of proteins that are specific to this species. However, many of these genes were not found in the genetic map of *S. mansoni*[16,18] and they do not contain protein domains traceable at Pfam [60]. BLAST searches against the non-redundant (nr) NCBI database detected a few non-*Schistosoma* proteins as significant hits that were annotated as hypothetical in all cases. For these reasons we rather believe that these sequences correspond to spurious predictions. Further analyses will be conducted in the future in order to confirm or refute this hypothesis.

Among the most significant protein expansions in *S. mansoni* we identified tetraspanins, fucosyltransferases, venom allergen-like proteins (SmVAL), tegumental-allergen-like proteins (SmTAL), leishmanolysins, and elastases, which were previously proposed as drug targets, once they can be related to morphological or physiological specificities of this parasite [5,20,61-65]. In these cases, the protein family membership ranged from 6 to 23 paralogs encoded in the parasite’s genome.

**Figure 4:**
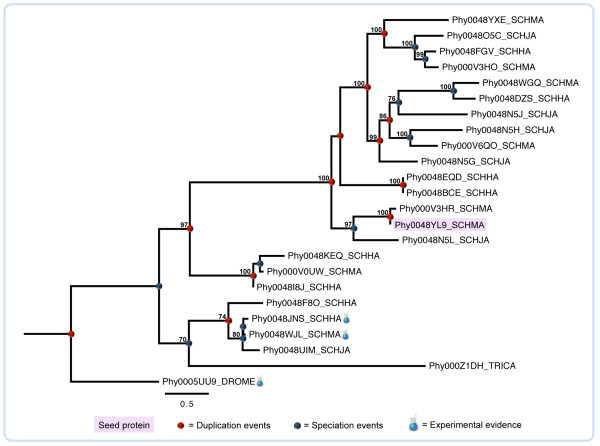
**Phylogenetic relationships of schistosome lineage-specific duplicated tetraspanins.** Analysis was performed with trimmed sequence alignment by using the maximum likelihood method as implemented in PhyML. Best fit model (WAG) and support values for each node were estimated by the Akaike Likelihood Ratio Test (aLRT). Sequence labels follow the PhylomeDB internal identifier. For details, see supplementary data (Additional file 1 Table S3).

Tetraspanins are small proteins with four transmembrane domains involved in the coordination of intra and intercellular processes, such as signal transduction, cell proliferation, adhesion, and migration, cell fusion and host-parasite interactions [66,67]. The function of schistosome tetraspanins are not completely understood, but cell-cell interactions and maintenance of cell membrane integrity might be performed by these proteins as well as they can be receptors for host ligands, acting on immune evasion [61]. The suppression of two tetraspanin genes (*Sm-tsp-1* and *Sm-tsp-2*) by RNA interference in mice also suggests that these proteins play important structural roles in the parasite’s tegument, being a good target for anti-schistosomal vaccine [68]. Figure 4 illustrates an example of tetraspanin lineage-specific duplications. In this case, the number of homologs in the three Schistosoma species varies from six to eight. Tree topology shows distinct well-supported clades suggesting that structural and/or functional variants might be present. Three proteins in this dataset have experimental evidence: Phy0048JNS_SCHHA (Q26499), Phy0048WJL_SCHMA (P19331), and Phy0005UU9_DROME (O46101) [49,69,70].

Venom allergen-like proteins (SmVAL), also called sperm-coating protein-like (SCP-like), are structurally related proteins members of the SCP/TAPS family. In Platyhelminthes, these proteins have been linked as potential modulators of immune function and components of sexual development [71]. Although the specific function of each SmVAL family member is unknown, there is evidence suggesting potential roles in larval penetration, host immune response modulation, and adult worm development [63,71]. Furthermore, analyses of SmVAL transcripts demonstrated that the corresponding genes are upregulated in infective stages of the parasite, highlighting SmVAL proteins as candidates for novel vaccine strategies [71,72].

Fucosyltransferases are enzymes that catalyses the fucose transfer from the donor guanosine-diphosphate fucose to different acceptor molecules such as oligosaccharides, glycoproteins, and glycolipids [73]. In schistosomes, fucosyltransferases are involved in producing immunomodulatory epitopes during infection, granuloma formation, egg/endothelium interactions, and were previously highlighted as anti-schistosomal candidates [63,74].

Tegumental-Allergen-Like proteins (SmTALs) are members of a protein family present in parasitic Platyhelminthes [64,75]. These proteins are located inside the tegument and have different life-cycle expression patterns [64]. The tegumental protein Sm22.6 is considered the main target for human IgE in *S. mansoni* and human IgE response against this protein is associated with the development of age-dependent partial immunity to *S. mansoni* infections in endemic areas [64,76].

Leishmanolysin, (also called invadolysin and SmPepM8), is a major surface protease member of the metallopeptidase M8 family. This protein can perform activities in schistosomes similar to those performed in *Leishmania* where these proteins are involved in different types of processes like degradation of the extracellular matrix and inhibition or perturbations of host cell interactions [63,72]. In turn, elastases are serine proteases that in schistosomes play a pivotal role in the penetration by cercariae of host skin to initiate infection. Recent studies have also revealed that these proteases can be employed by schistosomes to overcome or evade the host immune response [77,78]. Members of *S. mansoni* peptidase families such as leishmanolysins, cercarial elastases, and cathepsin D proteins were subjected to a detailed study in respect to their domain architectures, functional properties, and evolutionary relationships as described elsewhere [65].

Another specific feature of schistosomes is related to their tegument. Distinct from nematodes, which have a cuticle covering and protecting the organism body, schistosomes are covered by a living syncytium bounded by a complex multilaminate surface, which undergoes several adaptations soon after infection is initiated [79-81]. The external double membrane plays a crucial role in host-parasite interactions, being responsible for diverse mechanisms of survival [19,82,83]. The development of a tegument, highly specialized and resistant to immune damage, was accompanied by evolutionary adaptations, for example, the expansions of other protein families encoding annexins, cadherins, and innexins.

Annexins are widely distributed in eukaryotes performing a broad range of important biological processes related to tegument membrane [84-86]. In schistosomes, annexins appear to be involved in parasite’s stability protecting against immune attack by the host as well as against structural breakdown [85,86]. Cadherins are adhesion molecules that mediate Ca^2+^-dependent cell-cell adhesion and whose duplication events happened probably in parallel to the advent of a third germ layer in flatworms [5,87]. Innexins are components of gap-junction proteins, the intercellular channels that allow for the exchange of ions and other small signal molecules [88,89]. In *C. elegans*, innexins have been implicated in different processes like electrical coupling between pharyngeal muscles, calcium propagation in the gut, gap junction-mediated oocyte, and sensory neuron identity [89].

In summary, we identified that approximately 45% of the *S. mansoni* predicted proteins that were covered by this phylogenomic analysis have, at least, one paralog encoded in the parasite genome that might have arisen by gene duplication events that occurred after its divergence from other selected taxa (Additional file 1 Table S3). In other eukaryotic genomes this value ranges from 30 and 65% [90], whereas in *C. elegans* this value is equal to 49% [91].

Altogether, the present results indicate that besides the exploitation of host endocrine and immune signals, the parasite genome exhibit multiple events of gene duplication which may be, at least partially, an adaptive response related to the parasitic lifestyle. These expansions probably reflect the intriguing complexity of evolutionary events that happened over time, resulting in important characteristics in schistosome’s biology with consequences to the disease it causes. Taking into account the host environment and the selective forces that it imposes to a parasite, the phylogeny of host(s) and parasite(s) are probably closely related, once this coevolution will be responsible for the continuity or elimination of such an interaction. Nonetheless, previous empirical experiments involving schistosomes and the intermediate host provide further support to suggest the potential for host-schistosome coevolution [92].

In this context, it is important to analyze the evolutionary history of protein families during screening for potential targets for drug and vaccine development. Incorporating the evolutionary perspective in drug development studies can improve our understanding regarding drug resistance and effectiveness, as well as to guide new strategies of drug discovery. Gene duplication events as well as adaptive evolution should be considered during this process, since an anti-parasitic drug could bind a single protein or in all proteins encoded by a multi-gene family [93]. As a consequence, therapies which target a subset of genes that arose by duplication may not be effective at low doses. To solve this problem, the drug's effectiveness can be increased when a single-copy gene is targeted and its function is inactivated causing complete perturbation of a vital pathway [93,94].

## Conclusions

Through a systemic approach, we may accelerate the advance towards the understanding of schistosomiasis, its etiologic agents, and host-parasite interactions, optimizing the discovery of therapeutic targets to the development of new drugs and vaccines. Besides promoting a significant improvement in the functional annotation of the *S. mansoni* predicted proteome, our approach provided relevant information about the parasite’s genome evolution such as the identification of gene duplication events and expanded protein families, supplying important information regarding the mechanisms involved in *Schistosoma*’s genome evolution. Among the parasite paralog groups, we identified proteases, tetraspanins, fucosyltransferases, venom allergen-like proteins (also called as SmVAL or SCP-like), and tegumental-allergen-like proteins (SmTAL) that may be related to morphological or physiological specificities of this parasite. In addition, we strongly believe that the *S. mansoni* phylome data will pave the way for other, more detailed analysis, such as those that have been already performed on expanded peptidases families [65].

One of the remaining challenges is to understand which evasion strategies enable this parasite to survive for years in a potentially hostile environment, protected from the host immune system action and/or actively making the host response ineffective. Different mechanisms may be involved in these processes, including the generation of variant proteins by expression of micro-exon genes (MEG), which have been pointed as a potential strategy [94], and small non-coding RNAs which perform many essential regulatory functions [95].

Insights obtained through this phylogenomic approach will help us to guide forward genetic approaches to better understand the host-pathogen relationships toward to the elucidation of novel drug targets and vaccine candidates urgently needed to reduce the morbidity and mortality caused by schistosomiasis worldwide. Continuing this work, a comparative analysis involving genomic, transcriptomic, and proteomic data from other helminth species as *Taenia solium*, *Echinococcus multiloculares*, *Echinococcus granulosus*, *Fasciola hepatica*, other parasites, and vectors will provide valuable information from a system-wide perspective of a broad range of organisms, improving our understanding regarding the parasitic lifestyle.

## Methods

### Organisms and sequence data

Predicted proteomes from 13 fully sequenced eukaryotic genomes were downloaded from JGI Genome Projects, SchistoDB, Quest For Orthologs, WormBase, BeetleBASE, and FlyBase (Table 1). The taxon sampling was selected according to the availability of the predicted proteomes and based on the phylogenetic position of each species. The comprehensive taxa selected cover important evolutionary innovations making this dataset set especially suitable for addressing the evolutionary innovations in schistosomes in the context of metazoan evolution. Model organisms were also included to provide functional annotations that could be potentially transferred to *S. mansoni* homologous proteins.

### Phylome reconstruction

To reconstruct the complete collection of phylogenetic trees for all *S. mansoni* proteins and their homologs in other 12 fully sequenced organisms (Table 1), we used a similar automated pipeline to that described earlier for the human proteome [39] (Figure 1). A local database was created containing data from the *S. mansoni* proteome and those of 12 other completely sequenced genomes/proteomes. Alternative splicing products and identical sequences from the *S. mansoni* proteome were filtered out. For each protein encoded in the *S. mansoni* genome (“seed”), a Smith-Waterman search [96] (E-value ≤ 10^-5^) was performed against the above mentioned database to retrieve proteins with significant sequence similarity. Sequences that aligned with a continuous region longer than 50% of the query sequence were selected and aligned using MUSCLE 3.6 [40], MAFFT [41], DIALIGN-TX [42], and M-Coffee [43] with default parameters. Positions in the alignment containing a high number of gaps were eliminated using trimAl [44], with a consistency cutoff of 0.1667 and a gap score cutoff of 0.1. Neighbor-joining trees were derived from the trimmed alignments using *scoredist* distances as implemented in BioNJ [45] and maximum likelihood trees were obtained as implemented in PhyML using the NJ tree as a starting point [46]. For each “seed” protein phylogenetic reconstruction, we tested four different evolutionary models (JTT, WAG, BLOSUM62, VT, LG, CpREV, and DCMut). In all cases a discrete gamma-distribution model with four rate categories plus invariant positions was assumed, the gamma parameter and the fraction of invariant positions were estimated from the data. Tree support values were computed by approximate likelihood ratio test (aLTR) as implemented in PhyML [46,97]. The evolutionary model best fitting the data was determined by comparing the likelihood of the used models according to the Akaike Information Criterion (AIC) [98].

### Prediction of homology relationships

To derive orthology and paralogy relationships among *S. mansoni* proteins and those encoded in the other genomes included in this study we used a *species-overlap* algorithm as described in [39] and as implemented in ETE (Environment for Tree Exploration) [99]. This algorithm uses the level of species overlap between the two daughter partitions of a given node to define it as duplication or a speciation event. The analysis starts at the protein used to generate the tree (“seed” protein) and runs through the internal nodes of the tree until it reaches the root. All the trees were rooted at the midpoint. If the two partitions share any species (if there is species overlap), the node is defined as a duplication node and the proteins are considered paralogous ones. Otherwise (if there is no overlap) the node is defined as a speciation node leading to orthologous proteins. Once all the nodes have been classified, the algorithm establishes the orthology and paralogy relationships between the “seed” protein and other proteins included in the tree according to the original definition of these terms [39,100]. A previous study has shown that the *species-overlap* algorithm produces reliable orthology predictions with higher sensibility than a strict reconciliation method [101].

### Orthology-based functional annotation

Based on the list of orthology and paralogy relationship we performed the transfer of functional annotation from each ortholog with known function to the *S. mansoni* “seed” proteins. To produce a confident set of functional predictions for *S. mansoni* proteins, we classified the list of orthologs in different subsets of orthology relationships (one-to-one, one-to-many, many-to-one, and many-to-many) between the *S. mansoni* proteins and the other proteins included in this phylome data. If no duplication has occurred since the speciation, the two genes form a one-to-one relationship. If subsequent duplications have occurred, other types of orthology relationships (one-to-many or many-to-many) were assigned [51]. One example of this classification is provided (Figure 2).

To further analyze such large data set, we built the SchistoPhylomeSQL a Structured Query Language relational database using MySQL as a database management system. This local database integrates information from PhylomeDB (http://phylomedb.org) and SchistoDB (http://www.schistodb.net). Access to the database was obtained using DbVisualizer version 7.0.5 (http://www.dbvis.com), a graphical user interface that allows developing and accessing database management system (DBMSs) in different operating systems. The SchistoPhylomeSQL database was the main resource for data mining in this work. Perl scripts and SQL queries were implemented to parse the text files and load them to the database.

### Detection of *S. mansoni* gene expansions

Using ETE [99], we analyzed the *S. mansoni* phylome data to identify protein families that were specifically expanded in the *S. mansoni* lineage since its diversification from the other metazoans (Additional file 1 Table S3). The duplication events defined by the *species-overlap* algorithm that only comprised paralogs from *S. mansoni* were considered lineage-specific duplications. In cases where the information extracted from more than one phylogenetic tree contained the same paralogous proteins, changing only the “seed” protein position, the data was filtered to obtain a non-redundant list of in-paralogs.

## Competing interests

The authors declare that they have no competing interests.

## Author’s contributions

LLS: carried out the phylogenetic and functional annotation studies, and drafted the manuscript. MM: performed the phylome reconstruction and functional annotation transfer. LAN: participated in the coordination of this study, and co-wrote this manuscript. AZ: wrote the Perl scripts for data manipulation and provided computational support for this study. TG: participated in the coordination of this study, supervised the phylome reconstruction, and co-wrote this manuscript. GO: participated in the design and coordination of this study, and co-wrote this manuscript. All authors read and approved the final manuscript.

## Supplementary Material

Additional file 1 **Table S1**: Functional prediction for the*S. mansoni* "seed" proteins. **Table S2**: Comparison between reviewed proteins with functional annotation at UniProt and GO terms transferred by phylogenomic approach. **Table S3**: Homologous proteins to the *S. mansoni* "seed" proteins. Click here for file
